# First-year implementation of mailed FIT colorectal cancer screening programs in two Medicaid/Medicare health insurance plans: qualitative learnings from health plan quality improvement staff and leaders

**DOI:** 10.1186/s12913-019-4868-5

**Published:** 2020-02-21

**Authors:** Laura-Mae Baldwin, Jennifer L. Schneider, Malaika Schwartz, Jennifer S. Rivelli, Beverly B. Green, Amanda F. Petrik, Gloria D. Coronado

**Affiliations:** 1 Department of Family Medicine, University of Washington, Box 354696, Seattle, WA 98195 USA; 2 Kaiser Permanente Center for Health Research, Portland, OR USA; 3 Kaiser Permanente Washington Health Research Institute, Seattle, WA USA

**Keywords:** Colorectal cancer screening, Fecal immunochemical testing (FIT), Implementation, Mailed screening programs, Medicaid, Medicare, Underserved, Health plan, Qualitative

## Abstract

**Background:**

Colorectal cancer screening rates remain low, especially among certain racial and ethnic groups and the uninsured and Medicaid insured. Clinics and health care systems have adopted population-based mailed fecal immunochemical testing (FIT) programs to increase screening, and now health insurance plans are beginning to implement mailed FIT programs. We report on challenges to and successes of mailed FIT programs during their first year of implementation in two health plans serving Medicaid and dual eligible Medicaid/Medicare enrollees.

**Methods:**

This qualitative descriptive study gathered data through in-depth interviews with staff and leaders at each health plan (*n* = 10). The Consolidated Framework for Implementation Research, field notes from program planning meetings between the research team and the health plans, and internal research team debriefs informed interview guide development. Qualitative research staff used Atlas.ti to code the health plan interviews and develop summary themes through an iterative content analysis approach.

**Results:**

We identified first-year implementation challenges in five thematic areas: 1) program design, 2) vendor experience, 3) engagement/communication, 4) reaction/satisfaction of stakeholders, and 5) processing/returning of mailed kits. Commonly experienced challenges by both health plans related to the time-consuming nature of the programs to set up, and complexities and delays in working with vendors. We found implementation successes in the same five thematic areas as well as four additional areas of: 1) leadership support, 2) compatibility with the health plan, 3) broader impacts, and 4) collaboration with researchers. Commonly experienced successes included the ability to adapt the mailed FIT program to the individual health plan culture and needs, and the synchronicity between the programs and their organizational missions and goals.

**Conclusions:**

Both health plans successfully adapted mailed FIT programs to their own culture and resources and used their strong quality management resources to maximize success in overcoming the time demands of setting up the program and working with their vendors. Mailed FIT programs administered by health plans, especially those serving Medicaid- and dual eligible Medicaid/Medicare-insured populations, may be an important resource to support closing gaps in colorectal cancer screening among traditionally underserved populations.

## Background

Colorectal cancer (CRC) screening decreases mortality [1], yet CRC screening rates remain well below the Healthy People 2020 target of 70.5%, especially among certain racial and ethnic groups, the uninsured, and those who are Medicaid-insured. In 2018, 65.2% of adults aged 50-75 in the United States (U.S.) were up to date on CRC screening, but falling far below this overall rate were American Indian/Alaska Native (56.5%), Hispanic (57.7%), Asian American (57.1%), and uninsured (29.7%) adults [2]. In 2015, the most recent year that these data are available, the rate of CRC screening among Medicaid-enrolled adults aged 50-64 was only 47.4% [3].

Mailing fecal immunochemical test (FIT) kits to patients who are due for CRC screening is an evidence-based strategy for increasing CRC screening that has been tested by clinics and health care systems [4–9] (e.g., CRC screening rates have increased from 26.3 to 50.8% in primary care clinics in a large nonprofit health care delivery system [8], from 17.8 to 56.5% in rural family physician offices [9]). Health insurance plans are now beginning to implement mailed FIT screening programs [10].

Critical to the expansion of such programs is the need for guidance on program implementation. Published literature describes barriers to and benefits of implementing clinic- and health system-based mailed FIT programs [7, 11, 12], but limited data exist on health insurance plan-based programs. In this study, we explore implementation challenges and successes specific to two health insurance plans (hereafter referred to as health plans) that serve enrollees in U.S. Medicaid and Medicare programs.

BeneFIT is an implementation study of two health plans’ mailed FIT programs. Each plan designed its own implementation model—one collaborative and one centralized [13]. Under the collaborative model, the health plan worked collaboratively with the health centers. The health plan coordinated and administered the mailing of FIT kits while partnering with health center staff to customize the materials and workflows. Under the centralized model, all elements of implementation were coordinated and executed at the health plan level. In this study, we used data from interviews with staff (e.g., administrative quality improvement leaders and program managers) of these two health plans and our research team to examine the successes and challenges the plans experienced in implementing their mailed FIT programs in the first year. This work provides critical information that can help health plans understand how to best launch mailed FIT programs.

## Methods

This qualitative descriptive study was embedded within the larger BeneFIT hybrid implementation-effectiveness study [14]. For the BeneFIT study overall, our research team recruited two health plans, one in Oregon and one in Washington state, willing to design and implement programs geared towards mailing FIT kits to the addresses of age-eligible health plan members overdue for CRC screening. To identify these health plans, research team members reached out to existing contacts across health plans serving Medicaid and Medicare enrollees in Oregon and Washington. This recruitment process is described in more detail in an earlier article [13]. The Washington plan is an organization that operates in multiple states and provides Medicaid and dual Medicaid/Medicare coverage for approximately 650,000 members in Washington state. The Oregon plan is an organization that provides Medicaid and Medicare (largely dual eligible Medicare-Medicaid) coverage for about 220,000 enrollees in Oregon. The health plans formed teams, consisting primarily of administrators and program managers within their respective quality improvement departments, to conduct this work. Health plans were given the freedom to tailor the mailed FIT program to their organizational resources and needs. One developed a collaborative model, the other a centralized model. The research team provided information and sample materials (e.g., pictorial/wordless FIT test instructions) based on prior mailed FIT programs, offered technical expertise as needed, and evaluated the program [15, 16]. Detailed information on the models and health plans’ motivations for implementing a mailed FIT program is provided elsewhere [13].

### Program implementation

The health plans developed their own program components, including workflows; relationships and protocols with print and FIT vendors; tools and data report tracking; mailed materials; staff, department, clinic, and provider communications; and staff training (Table 1). Although health plans had their own implementation strategies, many components were similar: both plans generated lists of members due for CRC screening; scrubbed the lists for ineligible members or for those whose health systems opted out; ordered FIT kits; sent the final list of eligible members, FITs, and mailing materials to a vendor that prepared and sent introductory letters and FITs; monitored and tracked FIT completion; and accepted claims from the lab that processed the returned FITs.

**Table 1:** BeneFIT Implementation Activities

Activities	Collaborative Model	Centralized Model
**Staff Preparation/Training**		
Training health plan staff	X	X
Training vendor staff	X	X
Training clinic staff	X	
**Determining Member Eligibility**		
Generating list of members due for CRC screening	X	X
Notifying providers and health systems about mailed FIT kit program		X
Processing provider and health system opt-outs		X
Coordinating health plan and contracted clinics/health systems to finalize member list	X	X
Scrubbing list of eligible members	X	X
Sending member list to vendor		X
**Introductory Letter and FIT Kit Mailing/Tracking**		
Ordering FIT kits	X	X
Distributing FIT kits and FIT kit mailing materials to vendor	X	X
Preparing and mailing introductory letter	X	X
Preparing and mailing FIT kits	X	X
Monitoring tracking system/sending regular activity reports to health plan/clinics	X	X
Calling members to ask if they received FIT and/or had questions; resending FIT kits if needed	X	X
Fielding incoming calls from FIT kit recipients	X	X
Reminding members to complete the FIT kit	X	X
**FIT & Results Processing**		
Clinics: Receiving returned FIT, placing lab order, and sending to lab for processing	X	
Lab: Receiving completed FITs and processing results	X	X
Lab: Notifying health plan about claims	X	X
Lab: Entering results in clinic EHR	X	
Lab: Sending results to print vendor		X
Print vendor: Sending results to clinics/providers		X
Print vendor: Notifying health plan about positive results		X
Care coordinators: Calling members with positive results to connect with provider		X
Providers: Reviewing results in EHR	X	
Clinics/providers: Notifying patients of results and following up on positive results	X	
**Evaluation/Program Administration**		
Processing insurance claims as needed	X	X
Conducting oversight meetings and conference calls		X
Reporting fiscal and budget-related information		X
Processing incentives	X	X
Reviewing and evaluating mailed FIT program results	X	X

Basic differences in first-year implementation, outside of the overall collaborative or centralized approach, included the delivery method for reminders following the mailing (mailed postcard vs. live phone call), type of FIT used (e.g., FIT used by individual clinics vs. a standard two-sample FIT across the health plan), where FITs were returned and how they were processed (clinic-specific labs vs. centralized lab and their processes), and how results were communicated to providers and patients (following standard clinic procedures vs. mailing hard-copy results to providers plus health plan staff delivering live calls to patients with abnormal test results encouraging them to follow up with their primary care provider).

### First year health plan programs

Both health plans used 2015 Healthcare Effectiveness Data and Information Set (HEDIS) criteria to identify plan enrollees not up to date on CRC screening. One health plan included six health centers with 28 clinics in the FIT program and sent 2812 introductory letters and 2650 FIT kits to eligible enrollees. Of those who were sent introductory letters, almost half were female (49.8%); 84.0% were Medicaid-insured, and 16.0% were dually insured by Medicare and Medicaid; and 69.2% were white, 17.6% non-white, and 13.2% missing race/ethnicity. The second plan included all but three of the 507 health centers with which it had contracts (the three were excluded because they had their own mailed FIT programs) and sent 8551 introductory letters and FIT kits. This enrollee population was 42.5% female; 79.0% Medicaid-insured, and 21.0% dually insured by Medicare and Medicaid; and 56.1% white, 17.7% non-white, and 26.2% missing race/ethnicity.

### Qualitative data collection

Research staff collected three types of data to ascertain the health plans’ experiences in implementing the mailed FIT program: field notes during planning meetings between the research team and the health plans, internal research team debrief interviews, and in-depth interviews with health plan staff (see Additional file 1 for the interview guide). Research staff trained in qualitative methods (JS, JR) attended phone meetings of the research team and both health plans to record field notes and document questions, concerns, barriers, and achievements as they developed and implemented their programs. The research team met twice for internal debrief interviews. The team was interviewed as a group by JS, using a structured guide that explored the Consolidated Framework for Implementation Research (CFIR) domains that the study team felt were most relevant to evaluation of the implementation of BeneFIT’s mailed FIT intervention: intervention characteristics, inner setting, outer setting, and process evaluation [17]. Information gathered from the meeting field notes and CFIR-based research team reflections was used to develop an in-depth interview guide for use with the health plan administrators and project managers who were involved in the design and implementation of the mailed FIT program. The health plan interviews were telephone-based, conducted by either JS or JR, lasted 45–60 min, and occurred between March and June 2017, roughly 6–9 months after the start of implementation. Along with questions specific to the CFIR domains, interview questions explored topical areas such as: description of implementation activities; challenges to and successes achieved in implementing the mailed FIT program; member and provider reaction/feedback; observed strengths and weaknesses of the program model; reaction to available program results; and reflections on improvements. The Human Subjects Division of the University of Washington reviewed and approved all interview procedures and materials.

### Analysis of qualitative data

The health plan staff interviews were the primary source of information on challenges and successes in the first year of program implementation. Interviews were audio-recorded and transcribed for content analysis [18–20]. The research team’s qualitative staff (JS, JR) followed an iterative content analysis approach, developing a coding dictionary following review of a subsample of health plan interview transcripts. Aided by a qualitative software program, Atlas.ti [21], staff applied codes to each transcript and met regularly to discuss application of the codes and refinement of the coding dictionary. Coding discrepancies were discussed and reconciled, and newly identified codes were applied to any prior coded transcripts. When coding agreement was reached, Atlas.ti query and retrieval functions were used to generate topical reports of coded text. These reports were reviewed by JR and JS to identify key content themes. Transcripts of debrief interviews with research staff using the CFIR domains were summarized in multiple reviews by JS, MS, and LMB. These, along with the field notes, were used to further check interpretation of the health plan interview analysis. This iterative process resulted in a set of summary themes that were reviewed with the larger research team. Any areas identified as unclear or uncertain were re-reviewed against raw interview transcripts, resulting in a set of refined, agreed-upon themes.

## Results

We completed five interviews with each health plan, with all staff instrumental to the design and execution of the mailed FIT program during the first year of implementation. Collaborative-model health plan interviewees included the chief medical director, clinical quality improvement manager, senior manager of primary care projects, population supervisor, and project manager. Centralized-model health plan interviews were conducted with the local chief medical officer, local director of quality, national vice president of quality, national director of clinical interventions, and national project manager. First-year challenges and successes are presented below. The findings, while guided by CFIR in the interviews to explore a full range of implementation concepts, are organized based on the experiences of the two health plans.

### Challenges to first-year implementation efforts

Both health plans experienced implementation challenges in five areas: program design, vendor experience, engagement/communication, reaction/satisfaction of stakeholders, and processing/returning of mailed kits (Table 2).

**Table 2:** Implementation Challenges Experienced in the First Year of BeneFIT

Key Themes	Theme Identified by Health Plans		Sampling of Illustrative Quotes
	Collaborative Model	Centralized Model	
**Program Design**			
More time-consuming and complex to set up/start than anticipated	X	X	*“At the very beginning we did have a few provider groups that opted out because they were already providing kits to their members. It was something we definitely had to deal with early on to weed out certain populations for provider groups who were already engaged in their own initiatives.” –* Centralized *“There was a question of what to do with patients who hadn’t yet been seen in their clinic, and every clinic kind of dealt with that separately. In the end we had one health center just not process a bunch of FITs that came in because they couldn’t get those patients seen and they didn’t want to process FITs for them – so that was a pretty big challenge.”* – Collaborative
Some health centers/provider groups less interested as prefer to “do own thing” or had other CRC screening plans	X	X	
Lack of accurate electronic health record data for member information (e.g., address)	X	X	
Unestablished patients created unprocessed kits, extra time in outreach, or differing approaches to resolve	X		
**Vendor Experience**			
Complexity and time working with vendors to get FIT kits ordered and distributed	X	X	*“I would say the biggest challenge was the ordering of the FIT – there was a shortage for a while…and we had the whole issue of expired kits…Also, physically getting kits to the vendor so they could mail them out took tremendous coordination, effort, emails, reminding and tracking.”* – Collaborative “*The largest barrier we encountered was around patients receiving the FIT kits from the vendor…it took longer than we anticipated for the pre-letter mailing to go out [from vendor]. And per our timeline we wanted the kits to be mailed about a week after that and unfortunately the kits were not mailed out [by vendor] until a month after the pre-letter went out.”* – Centralized
Delay in obtaining kits from vendor to mail them out (e.g., large quantity or out of date)	X	X	
Delay in vendor mailing introductory letter and kits		X	
Lack of sufficient oversight with vendor so difficult to know exactly how many reminder calls were being completed or if following script		X	
Current lab vendor requires a two-sample test which may be barrier to FIT completion for patients		X	
**Engagement and Communication**			
Lack of communication with other key departments (e.g., membership services) about mailed program so less able to address patient questions	X	X	*“The customer service people at [health plan] didn’t really know about the program and should have because they would get [member] phone calls – so we should have written something up for them beforehand*.” – Collaborative *“Once we started getting our first list in for members that needed to receive calls to make sure to follow-up with their provider on the positive screening, it was sort of a scramble to figure out who was going to make those calls. The department at the time of signing up for the BeneFIT project had staff to do it, but once we received the actual list they didn’t have time and staff - so that staffing barrier needed to be addressed*.” – Centralized
Lack of staffing for follow-up calls regarding positive results/initial staff designated for this work unavailable once call list was ready		X	
**Reaction/Satisfaction of Stakeholders (provider/health center/patient)**			
General patient resistance to completing a FIT and doing CRC screening	X	X	*“The delays with the timeline and vendor also impacted members who were thinking they were going to receive the kit a week after the letter. And when members didn’t receive it, well, that’s when the calls started coming in…[members]saying ‘I haven’t received it in the mail.’”* – Centralized *“There is still that resistance to using FIT out there in some areas…we had one health center group that had very little penetration of patients touched by the BeneFIT program, and [in those] we have a high percentage of clinics where provider groups are still pushing colonoscopy.”* – Collaborative
Patient calls asking about whereabouts of FIT due to delay of kit mailing		X	
Providers desiring response rates for their patients/teams but information not available at time of inquiry		X	
Provider/health center resistance to FIT screening in some locations	X		
**Processing/Returning of Mailed Kits**			
Returned FIT kits not always processed	X		*“A big challenge was making sure all the names and dates were on the collected samples so when sent to the lab we wouldn’t get any rejections…but there was a break in communication between the lab and the clinics. The lab would get the test but wouldn’t process it for one reason or another and they wouldn’t let the clinic know – so the clinic would never know, and the patient never knows…that kind of feedback loop was never closed.” –* Collaborative *“We’ve seen some results, but we’re not sure on what the true [return] rates are at this point. The vendor had some issues [delays]…and we have some claims we are waiting on, so we don’t know what the real improvement will be [yet].” –* Centralized
Lack of workflow/process to ensure returned FIT kits were properly labeled before going to lab for processing	X		
No system in place to inform patient their completed FIT kit was not processed due to an issue (e.g., mislabeling)	X		
Some health centers were lower performers due to staff turnover or longstanding operational issues	X		
Assessing fully kit return rate outcomes hindered by delays and lag in claims data (e.g., up to 6 months)		X	

#### Program design

Staff from both plans said they were surprised by the amount of time required and the complexity of setting up their mailed FIT programs, particularly in determining accurate eligibility lists and establishing workflows and vendor expectations. Both plans were also challenged by lack of accurate data on members, such as current addresses. Additionally, both plans had provider groups or health centers opt out due to their own CRC screening programs. A unique issue that arose for the collaborative-model health plan was unestablished members—those patients assigned to their clinic but not yet seen by a provider. Leaders in some health centers expressed concerns about receiving and processing a mailed FIT kit for patients who had not first established care. Addressing this issue of unestablished clinic patients required some health centers to spend additional time and create new workflows (i.e., sending promotional letters in place of a mailed FIT kit or making phone call attempts to reach patients and encourage them to establish care with the clinic).

#### Vendor experience

Staff from both health plans said the communication and time spent working with vendors to order and distribute the FIT kits were challenges, which resulted in a delay in mailing the kits. Those working under the centralized model experienced additional challenges, including: the required use of the laboratory’s two-sample FIT test, which may have created patient barriers to completion; the mail vendor sending the introductory letter and kit later than planned; and lack of oversight in ensuring vendors were making the expected number of reminder phone calls or properly following the reminder call script.

#### Engagement/communication

Both health plans experienced challenges in communicating with key departments (e.g., member services) about the mailed FIT program and in ensuring that patients were given the correct phone number and/or contact person when they had questions about the introductory letter or mailed kit. For the centralized-model health plan, vendor delays in mailing kits created additional staffing issues in conducting follow-up calls to patients with abnormal results (positive for microscopic blood), as the staff identified for this work were less available at the time follow-up calls were due.

#### Reaction/satisfaction of stakeholders

Staff in both health plans felt that overall, patient resistance to CRC screening and the “yuckiness” of stool-based screening posed a challenge. Centralized-model health plan staff also fielded calls from members wondering when they would receive their FIT kit as it had not arrived in a timely fashion following the introductory letter. Additionally, centralized-model providers wanted to know the response of and outcomes for their patients sent the mailed FIT, and this information was unavailable in real time. In the collaborative-model health plan, some providers continued to prefer colonoscopy over FIT as a primary screening method. Given this preference, plan leaders felt there might be less acceptability and potential “penetration” of the mailed FIT program in these clinics.

#### Processing/returning of mailed kits

At the time of the interview, centralized-model health plan staff expressed slight concern about not having complete data on first-year FIT completion rates due to both the delayed mailing timeline and lag in claims data availability, making it difficult to assess the progress and impact of the program. For the collaborative model, several FIT kit processing issues may have affected return rates in the first year. For example, some completed and returned FIT kits were not processed due to mislabeling, the health plan member not being assigned to a provider, or the lab vendor determining the kit was not properly completed (e.g., missing a collection date). There was a lack of standard workflow to ensure completed mailed FIT kits were properly labeled (e.g., calling patients who provided no collection date) before going to the lab for processing, and no system was in place to inform patients that their completed and returned FIT was not processed.

### Successes with first-year implementation efforts

The health plans described success in the same five areas in which they had challenges, along with four additional areas: leadership support, compatibility with the health plan, broader impacts, and collaboration with researchers (Table 3).

**Table 3:** Implementation Successes Experienced in the First Year of BeneFIT

Key Themes	Theme Identified by Health Plans		Sampling of Illustrative Quotes
	Collaborative Model	Centralized Model	
**Program Design**			
Flexibility for each health plan to be either centralized or collaborative	X	X	*“[Program] allows more people to be identified and screened…to really identify and get more people screened so we can move upstream any care that might be necessary for a member.”* – Centralized “*A lot of other metrics to move are ones where patients need to come in for a clinic visit and so [we] particularly like this strategy because it can be done outside of the clinic…and clinics were like, ‘we don’t have to do anything and you’re going to help us get more patients screened? Great! And clinics would sign on because the [mailing] effort was coordinated at [health plan].”* – Collaborative
Another avenue to screen patients outside of a clinical visit/decreases patient burden	X	X	
Partnership approach/design encouraged health center participation by reducing staff burden and cost to implement CRC screening on own	X		
Health centers involved in project had all used FIT previously	X		
Encouraging health centers to scrub or clean eligibility list prior to mailing to update screening or identify wrong address	X		
**Vendor Experience**			
Documentation level from Vendor has been helpful		X	“*We had great success with follow-up calls to our members and getting them appointments for positive screenings and get them into the office – that worked well*.” – Centralized *“What we learned with the ordering and processing of kits [with vendor] was a process…like just getting the workflow of the kits sent here directly to [health plan], instead of kind of being the middleman and just shuffling things around, and that seems to be working pretty good now.”* – Collaborative
Success completing follow up calls and making appointments for members with abnormal result		X	
Working through initial challenges to order and mail kits valuable	X		
**Engagement and Communication**			
Familiarity with health centers and strong relationships	X		“*The provider groups were informed well in advance on the project and were happy with the information…and another success is that we are getting more provider engagement around the intervention and understanding around what we are doing here in the quality department*.” – Centralized *“[Health centers] are invested in it, they are committed to it…they spread it to their teams. They had meetings and explained the [program] – they knew what was happening… we didn’t have any clinics take part that hadn’t already used FIT before in their clinics*.” – Collaborative
Staff at health centers knew what was happening regarding the program / and took work personally to work hard and achieve goal	X		
Provider groups and their teams were engaged and informed of program		X	
**Reaction/Satisfaction of Stakeholders (provider /health center /patient)**			
Positive reactions from health centers and providers (e.g., no complaints)	X	X	“*Members were very appreciative…We often don’t get a sense of member [satisfaction], but through this we were able to do a lot of member outreach after a possible positive result and actually speak to members…who were telling us about their experiences of screening.”* – Centralized “*Providers are very grateful that this is just another way that we can tap into getting them in for screening…and the resources required at clinic level were so low – it’s when the FIT comes back in where most of clinic resources are and they already staff that generally — so running the program at the clinic level was low enough that clinics weren’t saying they couldn’t staff it*.” – Collaborative
Positive reaction from patients (e.g., no complaints)/expressions of appreciation	X	X	
Some patients called in to share they had their colonoscopy or FIT completed	X	X	
Patients were appreciative of follow-up call after abnormal FIT result		X	
Health centers that participated experienced minimum time and staff burden	X		
**Processing/Returning of Mailed Kits**			
Results intriguing enough to continue program for second year	X	X	*“We are really pleased to see how many of our Medicaid members that we included in the intervention actually complete a kit. We’re usually very excited if we can get a very small percentage of our members to participate in an intervention that we target them for…. And then with our Medicare population we had an [increase] of members complete the kits – so we are very excited about the outcomes that we did receive.”* – Centralized “*We are pleased with the partnership model showing success…actively sending out kits to members through the BeneFIT program, you can actually see [clinic] numbers are incredibly higher. For some it’s almost doubled from what they were normally doing.”* – Collaborative
Act of trying it for the first year a success in and of itself: establish workflows and address challenges as move into second year	X	X	
Access to follow-up colonoscopy for abnormal results going well	X	X	
More members are being screened for CRC with mail out program than previously	X	X	
Return rates for Medicare and Medicaid members better than in past mailed efforts and seems promising		X	
CRC screening rates were higher in many health centers participating in program and improved from prior years	X		
**Leadership Support**			
Support and strong champion to lead program at health plan level	X	X	“*It was helpful to have leadership buy-in from the beginning – to have [multiple health plan] leaders a part of the process early on and heavily involved at the very beginning – that’s important for operating any large intervention.”* – Centralized “*[Health plan] has been sold on this project from the beginning…and there is also medical collaboration too. Medical directors have been meeting with provider groups and giving presentations on the importance of FIT as a method of screening*.” – Collaborative
Medical directors at health centers promoting FIT and idea of mailed FIT	X		
**Compatibility with Health Plan**			
Matches well with organization’s mission and goals	X	X	*“From a program perspective I think it gets to what is needed and necessary for improving access to the tests and for more testing to be completed – so as a program it runs well.”* – Centralized *“This whole approach of collaboration…is another big driving factor for this… [Health plan] has a really strong ethos of working with clinics to help their patients get healthier and to push out preventive care…[and] is known for working with their clinics in creative ways*.” – Collaborative
Project housed within group that measures how clinics/providers are doing on quality metrics, including CRC screening	X	X	
Organization known for working with their health centers in supportive and partnering ways	X		
**Broader Impacts**			
Program helps to increase patient engagement with their provider, the health plan, and their own health	X	X	*“It really did invigorate our member outreach…it has helped us with thinking about other outreach to members that might be successful*.” – Centralized *“This shows a path to improve colorectal cancer screening which is critical in and of itself. But importantly, what it’s saying is here is a model of how you can go about doing things – a model for expanding into other things outside of colorectal cancer screening*.” – Collaborative
Increased general awareness of providers and other staff regarding activities in the health plans’ quality program departments, and specific awareness about mailed FIT programs	X	X	
Provides a roadmap for addressing population health efforts via mail out programs for other care gap areas	X	X	
**Collaboration/Resources from Researchers**			
Helpful for health plans to learn from research staff, and learn how other plan is implementing the mailed FIT program (materials, design and delivery, etc.)	X	X	*“One reason we like this so much is because there is a really important part of having [the researchers]and all the information and data that’s being collected and presented to then inform beyond colorectal cancer screening.”* – Collaborative *“That partnership and seeing what others have done successfully in the field helped us improve our intervention…and it was really nice to include the [wordless instructions] with the kit…for those members who might have had a difficult time reading…”* – Centralized
Ability to use wordless instructions developed by researchers for previous project		X	

#### Program design

Staff from both plans appreciated the flexibility to adapt core components of the mailed FIT program. They also viewed the program as an additional screening avenue outside of the clinical visit that could potentially decrease patient screening barriers (e.g., travel, time) while improving screening rates. Collaborative-model health plan staff felt their partnership approach was a benefit in that it encouraged health center participation by tailoring some components (e.g., introductory letter, type of FIT) to each health center’s preference while alleviating both cost and work burden through centralization of key pieces such as mailing of kits. Collaborative-model health plan staff believed that encouraging health centers to choose their own workflow practices—such as reviewing eligibility lists for prior CRC screening or conducting reminders to encourage FIT returns—was also a successful element of their program design.

#### Vendor experience

While the centralized-model health plan had some oversight challenges with its vendor, staff also felt that the tracking documentation they received regarding mailings and reminder calls was helpful during the early phase of implementation. They also felt that the planned workflow of having health plan staff make outreach calls to members who received abnormal FIT results was a success. Collaborative-model health plan staff felt the effort of managing delays from the vendor in ordering and mailing the FIT was in and of itself a first-year success.

#### Engagement/communication

Under the collaborative-model health plan, familiarity with and strong partnering relationships with the health centers were identified as a key driver of success. Additionally, clinic leads at the health centers were informed of and invested in the program, and actively communicated about the program with their staff, who were perceived as motivated and focused on achieving the goals of the program. Centralized-model health plan staff felt that they had successfully communicated with provider groups about the mailed FIT program.

#### Reaction/satisfaction of stakeholders

Both health plans received positive feedback from provider groups and health centers. Positive reactions from members were also noted by both health plans, e.g., members offered expressions of gratitude to staff, and some called to share that they had completed their screening. No provider group, health center, or member complaints were received by either health plan. Members of the centralized-model health plan expressed appreciation for the follow-up call they received explaining next steps after their abnormal FIT result. Within the collaborative-model health plan, health centers reported minimal burden on staff or time in implementing the mailed FIT program.

#### Processing/returning of mailed kits

A variety of successes related to processing and returning of the mailed FIT kits were identified by both health plans. First, implementation of the program helped to establish workflows and identify areas of improvement for the second year. Additionally, no issues regarding access to colonoscopy for an abnormal FIT result were reported in the interviews. Overall, leadership from both health plans felt the mailed FIT program was assisting their organizations in meeting CRC screening metrics and FIT completion rates were promising enough to continue for a second year.

#### Leadership support

Both health plans listed as successes strong champions within their organizations who endorsed and guided the mailed FIT program at the leadership level, as well as capable staff leading the day-to-day implementation. Collaborative-model health plan staff also felt it was beneficial that some of their health center medical directors actively promoted FIT as a primary screening method and supported the concept of a mailed FIT program.

#### Compatibility

The mailed FIT program was viewed by both health plans as matching well with their organizational mission and goals, including their desire to improve CRC screening rates for their membership. Additionally, both health plans had established quality improvement departments focused on programs to improve population-based measures that could house and execute the mailed FIT program. Collaborative-model health plan staff also felt the mailed FIT program aligned well with the plan’s history of working in partnership with its health centers and clinics to implement new and/or innovative programs.

#### Broader impacts

Both health plans identified three broader impacts they believed were created by participating in the BeneFIT mailed FIT program. First, leaders felt the program helped to enhance patient involvement not only with their own health, but also with their provider, health clinic, and health plan. The program was also viewed as increasing provider and clinical staff awareness about the kinds of activities undertaken by the health plans’ quality departments, including about how a mailed FIT program works. Collaborative-model health plan leaders felt health center staff gained knowledge about CRC screening in general and learned specific skills such as how to identify poorly labeled returned kits and correct them. Finally, staff from both health plans believed participation in the mailed FIT program offered a roadmap for how to address other care gaps using a population-based mailing approach.

#### Collaboration with researchers

Both health plans found it helpful to draw upon the expertise of the research staff as well as to learn about the other health plan’s model and how it was addressing implementation challenges. Centralized-model health plan leaders identified the ability to use a set of pictorial/wordless instructions with their FIT kit mailing as a success [22].

Based on their experiences in the first year, the health plans made suggestions for others to consider during the early phases of planning a similar effort. Table 4 highlights their advice, categorized into four key areas: engagement, planning, member eligibility, and FIT mailing/tracking/processing. Regarding engagement, both health plans emphasized the importance of early and continuing support and buy-in from all stakeholders, such as health plan leadership and staff, clinics and provider groups, and vendors. With planning, both health plans highlighted the importance of allowing at least 6 months of planning time prior to implementation to establish protocols and vendor relationships. Additionally, both noted that adequate resources of all types (e.g., staffing, funding for the cost of kits, mailings, and reminders), along with adequate communication about the program to key departments (e.g., membership services), were critical to robust program planning and implementation. The health plans also underscored how working with vendors requires close, constant oversight and communication to ensure program components are both implemented within scope and well documented. Finally, each health plan identified specific advice in the areas of member eligibility and FIT mailing/tracking/processing according to their respective models. The collaborative-model health plan advocated the importance of cleaning the mailing list prior to mailing (e.g., removing inaccurate addresses), while centralized-model health plan staff suggested allowing provider groups or clinics the opportunity to opt in or out of the mailed program. Using the same FIT kit that individual clinics or provider groups used, and establishing protocols for accurately labeling completed FIT kits for processing, were emphasized by the collaborative health plan. The centralized health plan recommended that there be “standing” or automatic orders for the FIT kits for eligible patients (versus individually ordered by providers) so health plans can efficiently execute the mailing of kits.

**Table 4:** Lessons Learned/Advice Following the First Year of Implementation

Key Suggestions	Collaborative Model (*N* = 5)	Centralized Model (*N* = 5)
**Engagement**		
Obtain strong leadership support and buy-in at health plan level at the onset	X	X
Make sure to have all the appropriate health plan leaders/staff involved early in planning process	X	X
Make sure clinics and provider groups are onboard & engaged as partners early on in planning process	X	X
Determine best way to engage and partner with vendors	X	X
**Planning**		
Develop a timeline/allow for at least 6 months of planning prior to implementation to work out complexities	X	X
Ensure adequate staffing at health plan level to execute program, including a designated project manager	X	X
Ensure all staff are aware of the program and there is adequate staffing for taking member calls, checking that returned kits are properly labeled, etc.	X	X
Identify resources to pay for kits, mailings, reminders, etc.	X	X
Establish clear and adequate oversight with vendors	X	X
Determine early on the return on investment model to evaluate the program		X
**Member Eligibility**		
Outreach methods (mail, reminder calls, etc.) should match the patient population being targeted	X	
Scrub (clean) the list prior to mailing and remove those already screened, not established at a clinic, no address, or not a good candidate	X	
If opt-out approach is offered or requested, extra time will be needed to take out certain populations for provider groups/clinics		X
**FIT Mailing/Tracking/ Processing**		
Develop data tracking spreadsheets and a reporting infrastructure	X	
Make sure to have process for ensuring FIT kits are labelled correctly so they are processed	X	
Have health centers/clinics use same FIT to reduce complexities	X	
Access or create a tool that will allow for standing orders so health plan can order kits		X
Track ongoing challenges to rectify any barriers to make the process more efficient		X

## Discussion

This study found that a mailed FIT program can be adapted to the culture and needs of individual health plans. Both health plans received positive feedback about the program from providers and patients, and plan leaders felt the results were positive enough to continue the programs for a second year. In addition, they felt that the process of implementing the mailed FIT program resulted in expertise that could be transferred to other population health programs.

The two models shared several challenges that spanned two domains in the CFIR model: characteristics of the intervention and outer setting. Implementation of the program was more time-consuming than anticipated, and there were complexities and delays in working with vendors—and in one plan, conducting other program tasks. These findings are not surprising, given that this was the first time both health plans had attempted a large-scale implementation of a mailed FIT program.

### Individual plan successes and challenges

Successes and challenges unique to the individual plans were most closely aligned with the CFIR domains of outer setting and inner setting. Not surprisingly, collaborative-model health plan leaders reported many successes related to their partnership with health centers. For example, the health centers were actively engaged in identifying individuals eligible for the program, and thus had a strong awareness of the program. Both staff and leaders in the clinical setting were committed to the program and could actively promote its success. Health centers were able to tailor materials (e.g., introductory letter) and workflows to their local setting without the staff burden of mailing the kits. These successes are a result of a collective commitment to the program, to sharing responsibilities and resources, and to learning from one another throughout the implementation process. These qualities are all components of collaborating and building strong partnerships, [23–26] and pave the way for collective efforts. However, there were challenges specific to the sharing of responsibilities and resources between the health plan and individual health centers, including dependence on clinic processes, preferences, and staff availability. The lack of standardized processes for all health centers complicated program management, tracking, and monitoring.

Staff with the centralized-model health plan reported success in tracking the program and its results through their vendor, which allowed the plan to follow up with individuals with abnormal FIT tests. The health plan asked the vendor to provide weekly updates on FIT kit returns, and the vendor reported FIT kit results to both the plan and the member’s primary care provider. In this way, the health plan’s care coordinators were able to make calls encouraging individuals with abnormal FITs to schedule a follow-up visit with their primary care provider. These efficiencies are consistent with centralized systems that prioritize both standardization and flexibility in program features, since decisions are made by a core leadership team [27]. This centralized process was also associated with reported challenges, such as questions from providers and members about the project and lack of inclusion of the local clinics in promoting and administering the program. In addition, even the centralized program’s staff reported that staff support for adequately monitoring vendor activities was a challenge, as the quality improvement staff responsible for the program had many competing quality initiatives that vied for their time.

### Mitigating clinic-level challenges

Coronado et al. have previously described challenges to implementing a mailed FIT program in clinic settings, with the most commonly reported challenges being related to the CFIR domain of inner setting: difficulties in access and accuracy of electronic health record data on CRC screening, and the time burden on staff [11]. Transferring the mailing of FIT kits to the health plan clearly helps alleviate these burdens. Health plan staff used available claims data to identify potentially eligible members, and these processes are familiar to health plans and their quality improvement staff. Similarly, both health plans had prior relationships with vendors, and in the centralized-model health plan, a relationship with a lab that could supply thousands of FIT kits at one time. However, staff from both plans reported challenges in working with their vendors and noted that having designated personnel at the plan to set up and oversee the work was critical.

Clinics in our previous study [11] identified as a challenge the lack of timely or accessible data to assess the success of a mailed FIT program. However, for this study both health plans worked with vendors to develop methods for tracking early implementation status pertaining to FIT mailings and returns. For example, the centralized-model health plan’s vendor routinely provided a tracking and monitoring log with information on FIT returns to assess early program implementation, while collaborative-model project staff reviewed bi-weekly individual health center-level claims data on FIT completion as a means of assessing program status. Additionally, both health plans eventually had access to claims data that could be used to examine FIT kit returns in relationship to mailings.

Our results are limited by the inclusion of only two health plan programs. The plans developed different models for their mailed FIT programs, and we do not know the degree to which the differences in the models influenced implementation. Additionally, we interviewed a small number of individuals at the health plans. We did not directly solicit other stakeholder opinions (e.g., providers, patients). However, given that the research focus was on program implementation, all relevant stakeholders at each health plan participated in the interviews. Additionally, we solicited their understanding of patient and provider reactions. Another limitation is the possibility of researcher bias when collecting field notes (e.g., misinterpretation of a “success” or “challenge”). Our use of CFIR domains to shape the study’s interview guide ensured that we had a strong guiding framework to capture implementation challenges and successes. However, we did not systematically explore CFIR constructs in each interview, and thus have not used CFIR to organize our study results. We enlisted several measures to ensure credibility and trustworthiness of our interview findings [28, 29], including: consistent use of an interview guide, formal coding and content analysis by a trained qualitative methodologist, triangulation of findings through integration and comparison to other sources of data such as field notes and research staff debrief interviews, and “member-checking” our findings by sharing and reviewing them with health plan staff.

## Conclusions

This research demonstrates the feasibility of implementing mailed FIT programs tailored to health plans based on their resources and preferences, as well as the substantial successes and addressable challenges that health plans experience as they work to lower rates of CRC and cancer death among their members. The two health plans participating in this study successfully adapted the components of prior clinic- and health system-based mailed FIT programs [4, 6, 8] to their own culture and resources, and found that their programs were a good fit with their organizational mission and goals. The availability of quality management resources allowed the health plans to overcome the most commonly described challenges reported for clinic- and health system-based mailed FIT programs—the time needed to set up and administer the program. Even with these resources in place, however, challenges common to both health plans included spending more time than anticipated to set up the programs and to work with their vendors. As health plans increase their investment in addressing population health management, they can use the results and lessons learned from this study to optimize the design of their own mailed FIT programs to best match their own organizational structure, resources, culture, mission, and goals. Mailed FIT programs administered by health plans, especially those serving Medicaid- and Medicare-insured populations, are an important resource in closing gaps in CRC screening among traditionally underserved populations.

## Supplementary information

**Additional file 1.** BeneFIT Implementation Interview Guide. In-depth interview guide for use with the health plan administrators and project managers involved in the design and implementation of the mailed FIT program.

## Data Availability

Data sharing is not applicable to this article as no datasets were generated or analyzed during the current study.

## Abbreviations

CFIR: Consolidated Framework for Implementation Research
CRC: Colorectal cancer
FIT: Fecal immunochemical test
U.S.: United States
