# Supplementary material for: First-year implementation of mailed FIT colorectal cancer screening programs in two Medicaid/Medicare health insurance plans: qualitative learnings from health plan quality improvement staff and leaders
Source: BMC Health Serv Res. 2020 Feb 21;20:132. doi: 10.1186/s12913-019-4868-5 (PMC7035739; doi:10.1186/s12913-019-4868-5)
Supplement: Supplementary file 1 — Additional file 1. BeneFIT Implementation Interview Guide. In-depth interview guide for use with the health plan administrators and project managers involved in the design and implementation of the mailed FIT program. [file 12913_2019_4868_MOESM1_ESM.docx]

**Implementation Interview Guide for BeneFIT’s Mailed FIT Colorectal Cancer Screening Programs in Two Medicaid/Medicare Health Insurance Plans**

1. **About You** *[ask if not someone interviewed previously at baseline]*
2. First, we'd like to learn a little about you. I’d appreciate it if you could give us a few words about your professional background?
3. How long have you been with [Health plan name]?
4. Would you please describe your role and various activities at [Health plan name]?
5. **Background History**
6. What is your familiarity with the BeneFIT program? Please describe.
7. How would you describe your role or involvement in the BeneFIT program to date?
   1. How long have you been in this role?
   2. Did anyone else fulfill this role for BeneFIT prior to you? [*Probe for turnover in roles, and if so, how and why this transition emerged?*]
8. What other staff or individuals have been involved in the implementation activities of BeneFIT? Please describe who they are and their role.
   1. Have any of these staff changed over time since the project started?
   2. Have any of the functions/activities of these other staff changed over time?
9. What factors, in your mind, have motivated your [Health plan name] to become [or remain] involved in the BeneFIT program? [*Ask open ended first, then probe on such things as*: *Belief in CRC screening; Cost/Benefit of the program; Effectiveness of the program; Acceptability/Ease of delivering the program; Assistance with addressing health disparities; Leadership/Champion within organization; Other external influences like state/national policy/other*]
10. Overall, how important is improving CRC screening for your [Health plan name]? Please explain
11. **Implementation**
12. What major activities have each of you been involved in with the BeneFIT program at [Health plan name]?
13. Please describe the target population you chose to receive this mailed CRC intervention?
    1. What factors shaped this decision?
    2. How did you go about defining and identifying your target population?
    3. Any challenges regarding this choice?
14. Given the implementation efforts so far, what have been challenges that you have encountered? [*allow to be open ended response*]
15. What have been the challenges to implementing the following activities: [*probe on each*]
    1. Identifying eligible patients and updating any invalid addresses/chart scrubbing efforts
    2. Getting appropriate lists of patients to print/mail or lab vendors
    3. Educating/informing providers or staff about the program
    4. Lab unable to process FIT kits or discard them for some reason (reasons, estimates of numbers)
    5. Process for transmitting or receiving results from the lab to clinic or providers
    6. Process for communicating results to patients
    7. Follow up colonoscopy referral/documentation for positive results
    8. Other challenges?
16. What has helped [Health plan name] in implementing the following activities: (probe on each)
    1. Identifying eligible patients and updating any invalid addresses/chart scrubbing efforts
    2. Getting appropriate lists of patients to print/mail or lab vendors
    3. Educating/informing providers or staff about the program
    4. Lab unable to process FIT kits or discard them for some reason (reasons, estimates of numbers)
    5. Process for transmitting or receiving results from the lab to clinic or providers
    6. Process for communicating results to patients
    7. Follow up colonoscopy referral/documentation for positive results
    8. Other activities?
17. Overall, have there been any surprises?
    1. Did things go as you expected with the program? Why or why not?
18. **Facilitators & Barriers**
19. What factors do you believe have helped [Health plan name] **develop** the BeneFIT program so far?
    1. Resource support
    2. Legislation changes
    3. Changes to fecal test
    4. Key staff/prior similar workflows
    5. Vendor cooperation
    6. Lab cooperation
    7. Internal and external support
    8. Leadership support
    9. Other
20. What factors do you believe have helped [Health plan name] **implement** the BeneFIT program, so far?
    1. Resource support
    2. Legislation changes
    3. Changes to fecal test
    4. Key staff/prior similar workflows
    5. Tracking reports
    6. Vendor cooperation
    7. Lab cooperation
    8. Internal and external support
    9. Leadership support
    10. Other
21. What factors do you believe have been a barrier to [Health plan name] **developing** the BeneFIT program so far?
    1. Resource support
    2. Legislation changes
    3. Changes to fecal test
    4. Key staff/prior similar workflows
    5. Vendor cooperation
    6. Lab cooperation
    7. Internal and external support
    8. Leadership support
    9. Other
22. What factors do you believe have been a barrier to [Health plan name] **implementing** the BeneFIT program, so far?
    1. Resource support
    2. Legislation changes
    3. Changes to fecal test
    4. Key staff/prior similar workflows
    5. Tracking reports
    6. Vendor cooperation
    7. Lab cooperation
    8. Internal and external support
    9. Leadership support
    10. Other
23. **Staff & Patient Reactions to BeneFIT**
24. What have you heard about how providers have reacted to the BeneFIT program?
    1. What do you see as provider barriers to the program?
    2. What do you see as provider facilitators to the program?
25. What have you heard about how [Health plan name staff] have reacted to the BeneFIT program?
    1. What do you see as staff barriers to the program?
    2. What do you see as staff facilitators to the program?
26. What have you heard about how enrollees have responded to the BeneFIT program?
    1. What do you see as enrollee barriers to the program?
    2. What do you see as enrollee facilitators to the program?
27. **Successes of Program**
28. At this point in implementation, what types of successes have you observed with the BeneFIT program?
    1. Enrollee-related benefits
    2. Provider-related benefits
    3. Contracted clinic-related benefits
    4. Organizational-related benefits
    5. Improvements in CRC screening rates Other
29. What do you believe to be the strengths of the program? Why is that?
30. What do you believe to be the weaknesses of the program? Why is that?
31. **Other Organizational Factors**
32. How has the organization been handling communication about the decision to launch the program, how the intervention works, what’s been going on during implementation of the BeneFIT program? Do you feel this communication has been sufficient or not – please describe? What suggestions do you have, if any, for how communication could have been handled differently?
    - - - 1. Communication with care coordinators or call center staff
          2. Communication with [Health plan name] leaders
          3. Communication with other [Health plan name] programs
          4. Communication with providers
          5. Communication with [Health plan name]-contracted clinics
          6. Communication with enrollees
        1. How have other initiatives or efforts that [Health plan name] is involved with impacted the BeneFIT program?
           1. Anything else that may have impacted CRC screening rates during implementation of BeneFIT? [*e.g., direct mail FIT programs initiated by Medicaid or Medicare Health Plans, care management, other?*]
           2. Any awareness campaigns, competitions, or incentives offered during this time?
        2. Has the BeneFIT study impacted other programs or initiatives in your [health plan name]? Please describe.
33. **Sustainability/Lessons learned**
34. How confident are you that you will be able to implement the mailed FIT kit program next year? Why is that?
35. What other kinds of changes or alterations do you think you will need to make [or want to make] to the intervention so it will work effectively in your setting?
    1. Do you think you will be able to make these changes? Why or why not?
    2. How can the BeneFIT team at the University of Washington help you as you implement the mailed FIT program next year?
36. What advice would you provide to colleagues at other organizations looking to implement the BeneFIT program?
    1. What could [Health plan name] have done differently?
    2. What would you say are the key lessons learned at this point?
37. What are the most important tools and resources for implementing a program like this?
38. **Final Comments**
39. Is there anything else you would like to share about your role and activities with the BeneFIT program to date?
